# *Mazus sunhangii* (Mazaceae), a New Species Discovered in Central China Appears to Be Highly Endangered

**DOI:** 10.1371/journal.pone.0163581

**Published:** 2016-10-26

**Authors:** Tao Deng, Xiao-Shuang Zhang, Changkyun Kim, Jian-Wen Zhang, Dai-Gui Zhang, Sergei Volis

**Affiliations:** 1 Key Laboratory for Plant Diversity and Biogeography of East Asia, Kunming Institute of Botany, Chinese Academy of Sciences, Kunming, Yunnan, P.R. China; 2 Key Laboratory of Plant Resources Conservation and Utilization, Jishou University, Jishou, Hunan, P.R. China; The National Orchid Conservation Center of China; The Orchid Conservation & Research Center of Shenzhen, CHINA

## Abstract

*Mazus sunhangii*, a new species of Mazaceae from central China is described and illustrated based on evidence from morphology and molecular phylogeny. This new species is morphologically similar to *M*. *puchellus* and *M*. *omeiensis* but differs in erect habit, inflorescence position, leaf pattern and corolla color. Phylogenetic analysis based on four chloroplast DNA regions (*rbcL*, *rps16*, *trnL-F*, and *psbA-trnH*) identified the new species as the independent lineage sister to the other East Asian *Mazus* species. The new species is known only from a single location in Mt. Shennongjia area in northwest Hubei province, at the elevation of 760 m. The species grows on the limestone cliff, and, because a tourist arterial highway is located along this cliff, its habitat can be easily disturbed or destroyed. We propose that the only known species location is recognized as critical habitat (i.e., as the habitat required to ensure the persistence of a species) and the species listed as Critically Endangered based on the International Union for Conservation of Nature Red List Categories and Criteria B2a.

## Introduction

*Mazus* Loureiro is a genus of perennial or annual herbs distributed in East and Southeast Asia, Australia, and New Zealand [1–3]. *Mazus* formerly was a member of Scrophulariaceae sensu lato, but in more recent taxonomic treatments based on phylogenetic analyses it was included in Phrymaceae [4] or even recognized as a family Mazaceae [5]. *Mazus* is a natural, well-delimited group of plants having a distinctive corolla, with 2 longitudinal clavate hairs on its throat (which is reflected in the Latin name “*Mazus*”, from the Greek mazos) [6,7]. However, species delimitation in *Mazus* has been problematic, with the reported number of species ranging from 10 to 30 or even more species [7,8]. Lack of consensus on infrageneric taxonomy is due to high morphological variability within the genus, with no agreement on taxonomic value of diagnostic characters used to distinguish species (e.g. stem and leaf margin shape, leaf position on a stem, and presence of hairs on the ovary) [7]. The existing controversy on the genus taxonomy, as well as recent discovery of several new species in China and New Zealand [9–11] necessitate the genus revision. The genus revision is also needed because the previous taxonomic treatments were based almost exclusively on morphology and did not include robust analysis of molecular data.

In China, 25 species and three varieties have been reported, of which 19 species and varieties are endemic and geographically restricted, and central and southwest China is considered to be the genus primary center of diversification [1]. Mainly focusing on Chinese species, Yang (1979) recognized three sections (*Trichogynus*, *Mazus*, and *Lanceifoliae*) in *Mazus* and his section delimitation was based on such traits as stem shape and presence of hairs on the ovary [12]. Notably, 9 species representing all three sections can be found in the Shennongjia National Nature Reserve (SNNR) region, which is located in the Northwest of Hubei province, central China.

The SNNR has unique geographical location and complex topography and is the core area of the “Metasequoia flora” region [12], one of the diversity hotspots in China and worldwide [13,14]. This region harbors more than 3600 species (Dai-Gui Zhang, unpublished data), many of which are endemics and “fossil” plants, such as *Metasequoia glyptostroboides* Hu et Cheng [15]. Despite its rich species diversity, the Shennongjia Mountains is still botanically under-explored region with continuing discovery of new species [16] and even genera [17] of the Angiosperms.

In recently conducted floristic explorations of the SNNR, an unusual isolated population of *Mazus* caught our attention. Examination of the collected material revealed that plants of this population are morphologically different from all currently known species of *Mazus*. Further research performed on the new taxon and related *Mazus* species, including field and herbarium comparisons and phylogenetic analysis based on four chloroplast (*rbcL*, *rps16*, *trnL-F*, and *psbA-trnH*) sequence data, supported the status of the taxon as a new species, which is described herein.

## Materials and Methods

### Nomenclature

The electronic version of this article in Portable Document Format (PDF) in a work with an ISSN or ISBN will represent a published work according to the International Code of Nomenclature for algae, fungi, and plants, and hence the new names contained in the electronic publication of a PLOS ONE article are effectively published under that Code from the electronic edition alone, so there is no longer any need to provide printed copies.

In addition, new names contained in this work have been submitted to IPNI, from where they will be made available to the Global Names Index. The IPNI LSIDs can be resolved and the associated information viewed through any standard web browser by appending the LSID contained in this publication to the prefix http://ipni.org/. The online version of this work is archived and available from the following digital repositories: PubMed Central, LOCKSS.

### Ethics Statement

No *Mazus* species is included in any Eurasian or Australian official list of threatened plants. The field studies did not involve endangered or protected species and the detailed information (including GPS coordinate) on the location of our study is provided in S1 Appendix. No special permits were required for this study because all samples were collected with introduction letters of KIB (Kunming Institute of Botany, Chinese Academy of Sciences). Voucher specimens were deposited in KUN.

### Morphological Assessment

Morphology of the newly collected specimens was examined and compared to that of *M*. *omeiensis* and *M*. *puchellus* based on the specimens from PE and KUN, as well as fresh materials of these two species from the field. In total, 15 individuals of the new species from the SNNR were examined, and deposited in KUN.

### Assessment of Conservation Status

We estimated the population size in the field, identified the threat factors, and assessed the endangered category according to IUCN red list criteria [18].

### DNA Sequencing and Molecular Analyses

We used 27 samples representing 19 taxa of *Mazus*, including the possible new species in molecular analyses. *Lancea tibetica* and *Dodartia orientalis* of Mazaceae, as well as *Paulowina tomentosa* of Paulowniaceae were chosen as outgroup taxa based on Schäferhoff (2010) [19] and our unpublished data. Sequences for other related taxa were obtained from the GenBank (see S1 Appendix). A list of samples, voucher location and GenBank accession numbers are provided in the S1 Appendix.

Phylogenetic reconstruction was based on DNA sequence data obtained from four chloroplast genes (*rbcL*, *rps16*, *trnL-F*, and *psbA-trnH*). Total genomic DNA was extracted from dried leaves, using the Universal Genomic DNA Extraction Kit V. 3.0 following the manufacturer’s protocol. Multiple-sequence alignment was performed by MAFFT v.6 [20], using the default alignment parameters followed by manual adjustment in Se-Al v2.0a11 (http://tree.bio.ed.ac.uk/software/seal/), and gaps were treated as missing data.

Phylogenetic trees were constructed using maximum parsimony (MP), maximum likelihood (ML) and Bayesian inferences (BI). The MP analyses were conducted using PAUP* v.4.0b10 [21]. The most parsimonious trees were obtained with heuristic searches of 1,000 replicates, with random stepwise sequence addition, tree bisection-reconnection (TBR) branch swapping, and multiple tree option in effect, and saving 100 trees from each random sequence addition. Bootstrap values of the internal nodes were obtained with 1,000 replicates [22]. In each replicate, 10 random sequence additions were performed, followed by TBR swapping, keeping no more than 1,000 trees per replicate. Maximum likelihood (ML) analyses were run in RAxML GUI 1.3 [23], followed by 1000 replicates. Bayesian inference was implemented using MrBayes v.3.1.2 [24]. The best-fit models of nucleotide substitution for the individual data partitions were determined with jModeltest 2.1.3 [25] using the Akaike Information Criterion. TVM+G was identified as the most appropriate model for *rps16* and *trnL-F*, while TVM+I and HKY+G were the best models for *rbcL* and *psbA-trnH*, respectively. Those models were replaced by the GTR option in both RAxML GUI and MrBayes given that the selected models were not available for analyses in both softwares. Bayesian tree topology was determined from two independent Markov chain Monte Carlo (MCMC) runs of four incrementally heated chains. Runs were performed for 10 million generations with sampling of trees every 1000 generations. When the log- likelihood scores were found to have stabilized, a consensus tree was calculated after discarding the first 20% of trees as burn-in. The remaining trees were imported into PAUP* and a 50% majority-rule consensus tree was produced to obtain posterior probabilities (PP) of the clades.

## Results and Discussion

### Taxonomic treatment

**Fig 1:**
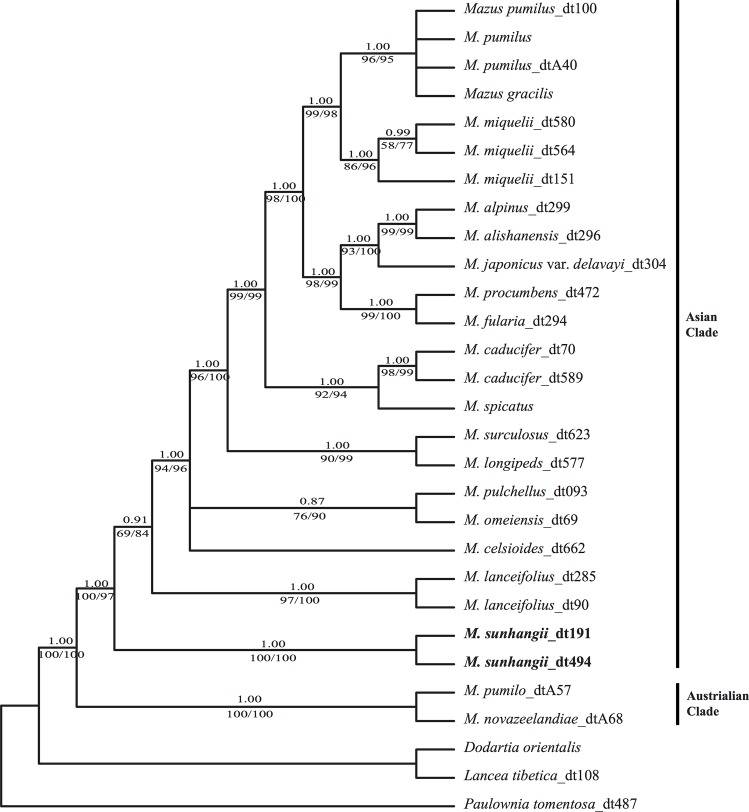
Bayesian consensus tree of *Mazus sunhangii* and related species. Numbers above branches indicate Bayesian posterior probability [PP], numbers below branches are maximum parsimony bootstrap [BP] and ML bootstrap (LP); the new species is shown in bold.

**Fig 2:**
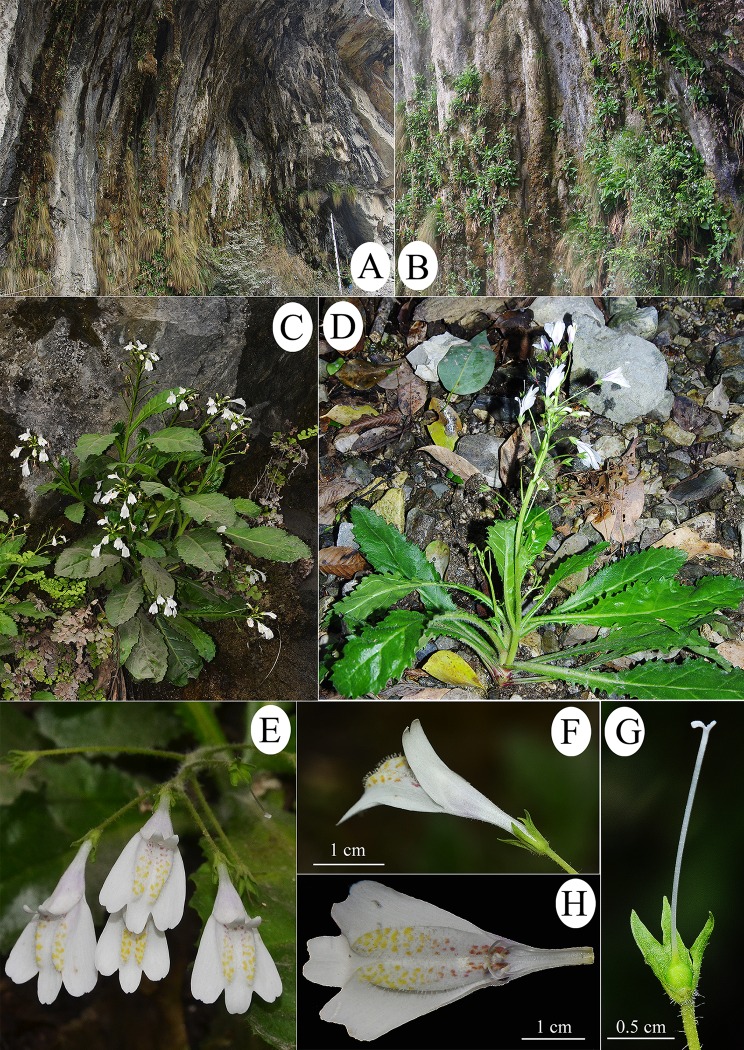
Images of living plants of *Mazus sunhangii* D. G. Zhang & T. Deng. **A,** habitat; **B,** population; **C**, habit; **D**, individual; **E,** inflorescence; **F,** flower; **G,** ovary and style; **H,** stamen.

**Fig 3:**
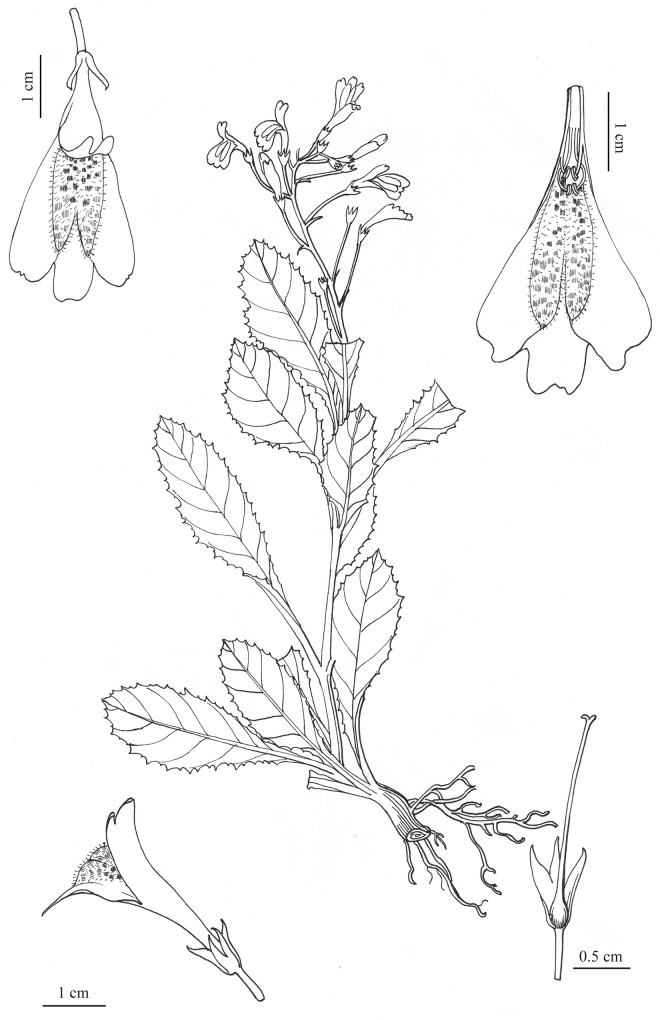
Holotype of *Mazus sunhangii* D. G. Zhang & T. Deng.

**Mazus sunhangii** D. G. Zhang & T. Deng, sp. nov. - 77157495–1 (Figs 1, 2 and 3).

#### Type

CHINA. Hubei Province: Shennongjia Forest District (SNNR), Xinhua town, Miaoerguan, 593 m, Long. 110.82E, Lat. 31.56N, 26 February 2012, flowering, *Dai*-*Gui Zhang*, *et al*. *4142* (holotype: KUN!; isotype: PE, A).

Perennial herb, 15–40 cm tall. Stems erect, 3–5 mm diameter. Basal leaves 3–5, leaf blade obovate-spatulate to oblong-spatulate, 8–15 × 4–6 cm, papery to thick papery, adaxial surface green, glabrous or with a few hairs, abaxial surface pale green, glabrous but only with sparse hairs on the veins, base tapering, margin irregularly pinnately serrate, acute at apex. Leaves margin usually uneven serrate, pinnately lateral veins 5–6 pairs, raised on abaxial surface. Stem leaves alternate or lower one opposite, 4–8, somewhat similar to basal leaves but smaller. Petiole 2–5 cm long, grooved.

Inflorescences terminal or terminal on lateral branches, ca. 15–20 cm long. Basal bracts bigger, similar to stem leaves, 2.5–5 × 1–3 cm, upper ones linear, 1–1.3 cm long. Pedicel 2–4 cm long, glabrous but with sessile glands. Calyx campanulate, ca. 8 mm long, green or brown-green, glabrous or hairy on margin, with prominent midribs on abaxial surface; teeth 3–4 mm, triangular-lanceolate, apex caudate.

Corolla 2–4cm long, white, but often opening pale yellow or flushed pale lavender then fading to white, flushed purple in throat and on proximal part of upper lobes, yellow on palate, glabrous apart from clavate hairs on palate and few scattered hairs on abaxial surface of lower lip. Tube 0.8–1cm long. Upper lobes 0.8–1.2 cm long, narrow-triangular; apex subacute, sometimes weakly obtuse or retuse. Lower lobes 1.5–2.5 cm long, usually rounded, sometimes square or rectangular, each apex usually retuse; outer two lobes spreading away from central lobe; palate comprising 2 longitudinal elevations extending from point of filament fusion to base of lower lobes; glabrous apart from clavate hairs on palate and few scattered hairs on abaxial surface of lower lip. Stamens 4, glabrous; filaments 5–7 mm long, curved; anthers dehiscing introrsely, positioned adjacent to corolla tube on upper lip. Ovary 2–3 mm long, glabrous, ovoid; style 7–9 mm long; stigma bilobed. Fresh fruit and calyx light green.

#### Phenology

Flowering occurs from February to April, and fruiting from April to June.

#### Distribution and habitat

**Fig 4:**
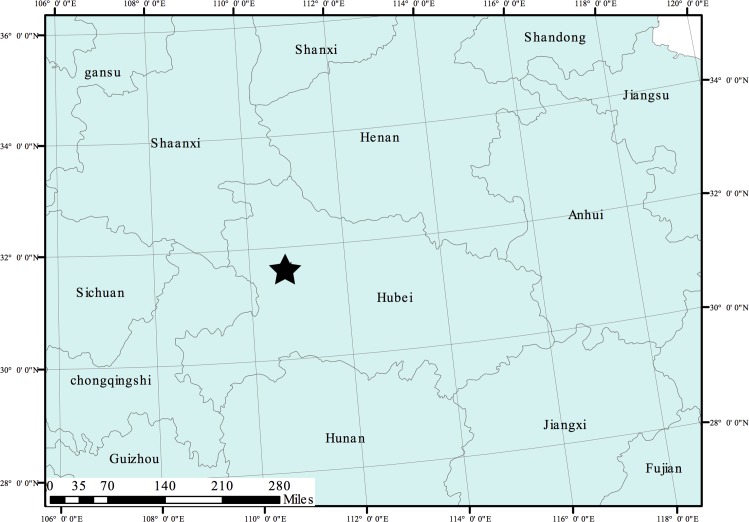
Distribution of *Mazus sunhangii* D. G. Zhang & T. Deng. The star indicates the type locality of *M*. *sunhangii*.

*Mazus sunhangii* is so far known only from a single location in the SNNR, the northwest part of Hubei Province, central China (Fig 4). The climate here belongs to subtropical monsoon, which is cool, foggy and humid (annual rainfall ca. 1770 mm). The only discovered population comprises less than 500 individuals growing along the steep moist limestone cliff up to the height of 200 m (Fig 2A and 2B). The total area occupied by the species is not more than 2 km. The other two species occupying the same habitat with *M*. *sunhangii* are *Eriophorum comosum* Wall. and *Adiantum capillus-veneris* L. The valley at the base of the cliff is covered by evergreen and deciduous mixed forest dominated by *Cyclobalanopsis gracilis*, *Platycarya strobilacea* and *Carpinus viminea*.

#### Etymology

The species is named in honor of Prof. Hang Sun, a Chinese botanist who made a significant contribution to our knowledge of the flora of China.

#### Additional specimen examined

China. Hubei province, Shennongjia Forest District (SNNR), Yangri town, Wugu Mountain, 110.01E, 31.00N, 450 m a.s.l., 4 Sep 2011, *D*.*G*. *Zhang & T*. *Deng 4600*, *T*. *Deng 2307* (KUN).

#### Species recognition

*Mazus sunhangii* is closely related to *M*. *pulchellus* and *M*. *omeiensis*. These three species share such features (which distinguish them from the other *Mazus* species) as plants 10–40 cm height, stem non-woody at base, large corolla (2–3 cm) and ovary glabrous. However, *M*. *sunhangii* differs from *M*. *pulchellus*and *M*. *omeiensis*in in a number of characters (Table 1). The most obvious differences are that *M*. *sunhangii* is an erect herb with 4–8 stem leaves, terminal inflorescences and white corolla while the latter are rosulate herbs with all leaves being basal and rosulate, with 1–5 scapes and red to purple and even dark violet corolla (Fig 3). Furthermore, the new species is also distinguished from *M*. *pulchellus* by its calyx lobes ca. 1/2 as long as tube and the rounded margin of corolla upper lips (Fig 3). In contrast, *M*. *pulchellus* has calyx lobes much shorter than tube, with fimbriate-toothed margin. Among these three taxa, *M*. *omeiensis* has short pedicels, 1–1.5 cm, whereas pedicels of *M*. *sunhangii* and *M*. *pulchellus* are much longer (up to 4 cm) (Table 1; Fig 3 and S1 Fig).

**Table 1:** Diagnostic morphological characters of *Mazus sunhangii* in comparison with other related *Mazus* species.

Characters	*M*. *sunhangii*	*M*. *pulchellus*	*M*. *omeiensis*
**Habit**	erect herb, sparsely villous	rosulate herb, densely white or rusty pubescent	rosulate herb, glabrous or sparsely villous
**Leaves**	with stem leaves (4–8) and basal leaves (3–4)	all basal, rosulate	all basal, rosulate
**Leaves texture**	papery to thick papery	papery	thick papery to subleathery
**Scapes**	no	1–5	1–4
**Pedicels**	to 4 cm	to 4 cm	1–1.5cm
**Corollacolor**	white	red, purple or dark violet	light blue-purple
**Upper corolla lips**	apexsubacute, sometimes weakly obtuse or retuse	apexfimbriate-toothed	apexsubacute

The leaves of *M*. *sunhangii* are similar to those of *M*. *pulchellus*, but are generally thicker and leaves abaxially light green, glabrous or only with sparse hairs on the lateral veins, which raised on abaxial leaf surface, whereas those of *M*. *pulchellus* densely hoar arachnoid tomentum (Fig 2 and S1 Fig; Table 1).

#### Molecular phylogenetic analysis

In our phylogenetic analysis, we used sixteen Asiatic taxa of the *Mazus* representing all three sections (the most representative sampling of the genus to date). The aligned matrix consisted of 3302 characters, of which 475 were variable and 272 were parsimony-informative. All three analyses (BI, MP and ML) produced trees with identical topology. The 50% majority consensus BI tree is presented in Fig 1.

Our molecular phylogenetic results disagree with the previous infrageneric classification of *Mazus* [12]. According to our results, there are two major lineages (Asian clade and Australian clade) in *Mazus*, matching their distribution in Australia and Asia (Fig 1). *M*. *sunhangii* is nested within the Asian clade with very high support (BS = 1.00, PP = 1.00, PL = 97). In this clade, *M*. *sunhangii* diverged first, and is sister to the clade with all other Asian *Mazus* species. A more representative sampling with improved estimation of phylogeny for *Mazus* may provide additional insights into the systematic position of the new species.

#### Conservation Significance

Despite extensive investigations in central China conducted by the authors after species discovery, the species has so far only been found in the area of Miaoerguan Mountain in the SNNR, in the southwest part of Hubei province, central China (Fig 4). The new species is probably calcicole. It appears to prefer shady and wet habitats with deep humus-rich soil. The only discovered population was growing on a single steep moist limestone cliff typical for the area ancient limestone mountains that are deeply eroded and dissected by deep river valleys.

The observed small population size and very limited area covered by the species warrant urgent defining the conservation status of *M*. *sunhangii* and its official legislation. We estimated the only discovered population to comprise less than 500 individuals. The species grows on a single limestone cliff c. 2 km long, and, because a tourist arterial highway is located along this cliff, its habitat can be easily disturbed or destroyed. The individuals growing close to the highway were found to be covered with a layer of dirt thrown by the cars from the highway. Road maintenance and possible upgrade in the future can cause disappearance of the only known population of the species. We propose that this unique location is recognized as critical habitat (i.e. as the habitat required to ensure the persistence of a species) and the species listed as ‘‘Critically Endangered” (CR Blab (v)+D) based on the International Union for Conservation of Nature Red List Categories and Criteria B2a.

## Supporting Information

S1 Appendix Taxa sampled and their GenBank accession numbers for the DNA sequences used in this study. (DOCX)

S1 Fig Images of living plants of *Mazus omeiensis* (A) and *M*. *pulchellus* (B and C).
(TIF)
