# Supplementary material for: Mazus sunhangii (Mazaceae), a New Species Discovered in Central China Appears to Be Highly Endangered
Source: PLoS One. 2016 Oct 26;11(10):e0163581. doi: 10.1371/journal.pone.0163581 (PMC5081197; doi:10.1371/journal.pone.0163581)
Supplement: S1 Appendix — (DOCX) [file pone.0163581.s001.docx]

**S1 Appendix: Taxa sampled and their GenBank accession numbers for the DNA sequences used in this study.**

| **Taxon** | **DNA no.** | **Voucher (Herbarium)** | **Locality** | **Coordinate** | **rps16** | **trnL-F** | **rbcL** | **psbA-trnH** |
| --- | --- | --- | --- | --- | --- | --- | --- | --- |
| *Mazus alishanensis* | dt296 | Sunhang11307 | Taiwan | 120.01, 23.00 | KX783501 | KX783520 | KX783481 | KX783459 |
| *M*. *alpinus* | dt299 | Sunhang11322 | Taiwan | 120.01, 23.00 | KX783500 | KX783519 | KX783480 | KX783458 |
| *M*. *caducifer* | dt070 | kifir037 | Anhui, China | 118.43, 29.56 | KX783497 | KX783516 | KX783477 | KX783455 |
| *M*. *caducifer* | dt589 | Deng688 | Anhui, China | 118.35, 29.54 | KX783506 | KX783526 | KX783487 | KX783465 |
| *M*. *celsioides* | dt662 | YIF-0093 | Xizang, China | 95.36, 29.59 |  | KX783525 | KX783486 | KX783464 |
| *M*. *fularia* | dt294 | Sunhang11248 | Taiwan | 121.01, 25.00 | KX783499 | KX783518 | KX783479 | KX783457 |
| *M*. *japonicus* var. *delavayi* | dt304 | Sunhang11459 | Taiwan | 120.01, 23.00 | KX783502 | KX783521 | KX783482 | KX783460 |
| *M*. *lanceifolius* | dt090 | 2011－11－17 | Hubei, China | 110.01, 31.00 | KX783491 | KX783510 | KX783471 | KX783447 |
| *M*. *lanceifolius* | dt285 | zdg4447 | Hubei, China | 110.01, 31.00 | KX783490 | KX783509 | KX783470 | KX783446 |
| *M*. *longipeds* | dt577 | Deng1941 | Guizhou, China | 104.85, 26.53 | KX783494 | KX783513 | KX783474 | KX783450 |
| *M*. *miquelii* | dt151 | Deng432 | Yunnan, China | 100.95, 21.86 | KX783495 | KX783514 | KX783475 | KX783451 |
| *M*. *miquelii* | dt564 | NK11186 | Yunnan, China | 100.01, 27.00 | KX783503 | KX783522 | KX783483 | KX783461 |
| *M*. *miquelii* | dt580 | Deng2165 | Yunnan, China | 104.45, 22.77 | KX783496 | KX783515 | KX783476 | KX783453 |
| *M*. *novazeelandiae* ssp. *novazeelandiae* | A68 | s.n. | New Zealand | 175.87, -41.57 | KX783489 | KX783508 | KX783469 | KX783445 |
| *M*. *omeiensis* | dt069 | nie1976 | Sichuan, China | 103.93, 29.87 | KX807203 | KX807208 | KX807209 | KX783449 |
| *M*. *procumbens* | dt472 | zdg6074 | Hubei, China | 110.01, 31.00 | KX783498 | KX783517 | KX783478 | KX783456 |
| *M*. *pulchellus* | dt093 | Deng2015 | Hunan, China | 109.72, 28.34 | KX783492 | KX783511 | KX783472 | KX783448 |
| *M*. *pumilo* | A57 | 2021829; Paget, s.n. | Australia | 174.82, -40.37 | KX783488 | KX783507 | KX783468 | KX783444 |
| *M*. *pumilus* | A40 | 535370; Li, 15512 | Yunan, China | 100.18, 26.97 | KX807202 | KX807207 |  | KX783454 |
| *M*. *pumilus* | dt100 | Deng403 | Hunan, China | 109.59, 28.34 | KX807201 | KX807206 |  | KX783452 |
| *M*. *sunhangii* | dt191 | zdg4142 | Hubei, China | 110.82, 31.56 | KX783504 | KX783523 | KX783484 | KX783462 |
| *M*. *sunhangii* | dt494 | zdg4600 | Hubei, China | 110.01, 31.00 | KX783505 | KX783524 | KX783485 | KX783463 |
| *M*. *surculosus* | dt623 | KUN0472212 | Yunnan, China | 100.07, 26.98 | KX783493 | KX783512 | KX783473 |  |
| *Lancea* *tibetica* | dt108 | Sunhang9875 | Yunnan, China | 99.91, 27.34 | KX807200 | KX807205 | KX783467 | KX783443 |
| *Paulownia tomentosa* | dt487 | zdg4529 | Hubei, China | 110.01, 31.00 | KX807199 | KX807204 | KX783466 | KX783442 |
