# Supplementary figures and images for: Mazus sunhangii (Mazaceae), a New Species Discovered in Central China Appears to Be Highly Endangered
Source: PLoS One. 2016 Oct 26;11(10):e0163581. doi: 10.1371/journal.pone.0163581 (PMC5081197; doi:10.1371/journal.pone.0163581)

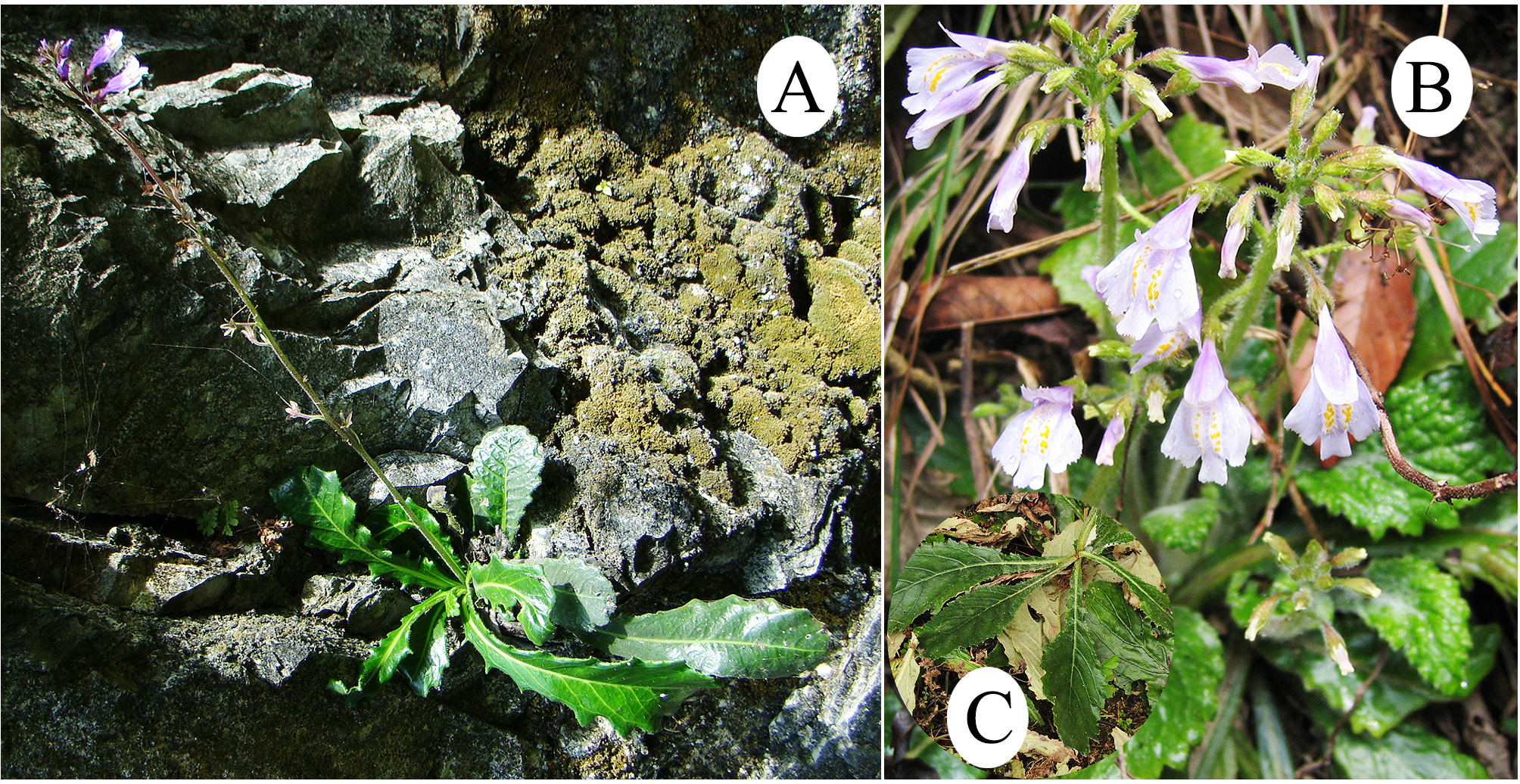

Supplement: S1 Fig — Images of living plants of Mazus omeiensis (A) and M. pulchellus (B and C). (TIF) [file pone.0163581.s002.tif]
